# Modeling of Flowering Time in *Vigna radiata* with Artificial Image Objects, Convolutional Neural Network and Random Forest

**DOI:** 10.3390/plants11233327

**Published:** 2022-12-01

**Authors:** Maria Bavykina, Nadezhda Kostina, Cheng-Ruei Lee, Roland Schafleitner, Eric Bishop-von Wettberg, Sergey V. Nuzhdin, Maria Samsonova, Vitaly Gursky, Konstantin Kozlov

**Affiliations:** 1 Mathematical Biology and Bioinformatics Lab, Peter the Great St. Petersburg Polytechnic University, 195251 Saint Petersburg, Russia; 2 Institute of Ecology and Evolutionary Biology, National Taiwan University, Taipei 106319, Taiwan; 3 World Vegetable Center, Tainan 74151, Taiwan; 4 Department of Plant and Soil Science, Gund Institute for the Environment, University of Vermont, Burlington, VT 05405, USA; 5 Program Molecular and Computation Biology, University of California, Los-Angeles, CA 90095, USA; 6 Theoretical Department, Ioffe Institute, 194021 Saint Petersburg, Russia

**Keywords:** flowering time, mung bean, artificial image objects, climatic factors, GWAS, convolutional neural network, random forest

## Abstract

Flowering time is an important target for breeders in developing new varieties adapted to changing conditions. In this work, a new approach is proposed in which the SNP markers influencing time to flowering in mung bean are selected as important features in a random forest model. The genotypic and weather data are encoded in artificial image objects, and a model for flowering time prediction is constructed as a convolutional neural network. The model uses weather data for only a limited time period of 5 days before and 20 days after planting and is capable of predicting the time to flowering with high accuracy. The most important factors for model solution were identified using saliency maps and a Score-CAM method. Our approach can help breeding programs harness genotypic and phenotypic diversity to more effectively produce varieties with a desired flowering time.

## 1. Introduction

Mung bean (*Vigna radiata* (L.) Wilczek), also known as green gram) shows a constant rise in production and a steady increase in importance in Asia and other developing areas. It is a self-pollinated short-duration crop that has received limited breeding efforts [1]. Mung bean is a valuable source of protein and essential micronutrients, such as folate and iron. It adds nitrogen to the soil, so it may provide additional income to farmers as a rotation crop, and it works well as a plant-based protein [2].

The size of the maung bean’s genome is relatively small, and the plant has a short life cycle. Diverse collections of mung bean have been organized in genebanks worldwide, such as the World Vegetable Center (Taiwan), the National Bureau of Plant Genetic Resources (India), the Institute of Crop Germplasm Resources (China), the Plant Genetic Resources Conservation Unit (USA), the genebank of the Commonwealth Scientific and Industrial Research Organization (Australia), Plant Genetic Resources Program (Pakistan), and the N.I. Vavilov All-Russian Institute of Plant Genetic Resources (Russia) [3,4,5].

The progress in marker-assisted breeding has been limited for mung bean [6] though several studies have been carried out recently [7] with important agronomic traits [4,7,8]. International studies revealed that time to flowering in mung beans is influenced by genetic [9] and environmental factors [10] together with genotype-by-environment (G × E) interactions [11,12].

The design of genome-wide association studies (GWAS) may become expensive and cumbersome, particularly in multi-enviromental setups. Thus, GWAS can be complemented by crop models that account for genotype-by-environment interactions. Machine learning (ML) methods are an alternative to classical statistical approaches to the analysis of genetic datasets. Random forest (RF) [13] has become a popular machine learning technique in recent years. The main idea of solving the problem is to train the model to predict the flowering time for a given set of single nulceodite polymorphisms (SNPs), then using various methods to find out which SNPs have the most significant impact on the prediction. In RF, multiple decision trees are built using offline sampling (bagging), and the method of random node splitting, the final classification, or regression results are obtained by voting or averaging. RF fits a model that includes all SNPs and does not require the assumption of inheritance patterns (e.g., additive, dominant, and recessive), making RF an attractive approach for complex traits. In recent years, a new area of machine learning research has focused on interpretability. These new methods can be used to rank genetic markers based on the strength of association with the phenotype in the model. The Gini index [14] and SHAP values [15] are widely used for this purpose, together with approaches such as the Boruta algorithm [16]. The application of these methods to the big datasets may be computationally expensive.

Various mathematical techniques are used for crop modeling, from linear regression to artificial neural networks [17]. Artificial image objects (AIOs) are a new concept for representation of genomic data. The advantages of AIOs are that their convenient straightforward visualization, compactness, and the ability to apply a vast number of techniques developed for image analysis and classification [18]. Particularly, convolutional neural networks (CNNs) were successfully used for classification of AIOs [19].

AIOs and CNNs possess calculation and visualization of the impacts of different factors to the final model solution. Recently, increasing attention has been drawn to the internal mechanisms of convolutional neural networks and the reason why the network makes specific decisions [20]. Several techniques have been introduced that include perturbation and backpropagation-based approaches [21], gradient-based algorithms [22], and class activation maps [20]. A saliency map represents the spatial support of a particular class in a given image [23]. Gradient-weighted class activation mapping (Grad-CAM) uses the gradients of any target concept flowing into the final convolutional layer to produce a coarse localization map highlighting the important regions in the image for predicting the concept [24]. Score-CAM, unlike previous class-activation-mapping-based approaches, eliminates the dependence on gradients by obtaining the weight of each activation map through its forward passing score on target class. The final result is obtained by a linear combination of weights and activation maps [20].

Developing on our previous studies for chickpea [25,26,27] and mung bean [28,29], we propose a new approach in this study that uses AIOs and CNNs to predict the time to flowering using SNP and climatic factors. The selected techniques include the application of computationally efficient algorithms for construction and optimization. The developed approach can help breeding programs harness genotypic and phenotypic diversity to more effectively produce varieties with a desired flowering time. The main contributions of this work are:

- The methodology is proposed that combines AIOs and modern ML algorithms,
- Using random forest and three feature importance measures, SNPs are selected that influence the time to flowering in the available *Vigna* dataset,
- A model based on AIOs and CNNs is developed for prediction of time to flowering in the available *Vigna* dataset using selected SNPs and climatic factors for only 5 days before and 20 days after planting,
- The impacts of genotypic and climatic factors and their combinations to the model solution are evaluated using two visual explanation methods.

## 2. Related Work

Crop models that utilize the information on molecular markers can be critically important tools [30]. Process-based models, such as DSSAT [31], AquaCrop [32], APSIM [33], and SSM [34] that account for weather conditions have been developed for different species including mung bean. The validation of obtained results was needed to assist breeding programs [35].

Machine learning methods have been used to increase the statistical power of GWAS [36], to detect epistatic interactions, to improve the polygenic risk estimate obtained using GWAS [37], and to post-process the results of GWAS analysis [38]. Recently, improved random forest (RF) [13] methods have been proposed and applied to GWAS, such as the T-Tree method and the ts-RF method [39], which optimize tree node separation rules; Szymczak (2016) [40] redefined the method for calculating importance scores; in [41], a hybrid RF algorithm was proposed.

Various methods for interpretation of CNN models have been proposed recently. Class activation maps provide a visual explanation for a single input [24,42] but are architecture-sensitive. Grad-CAM [24] and its variations, e.g., Grad-CAM++ [42], generalize CAM to models without global pooling layers. LayerCAM [43] may generate reliable class activation maps from a combination of class activation maps from different layers of the CNN.

## 3. Materials and Methods

### 3.1. The Overview

**Figure 1:**
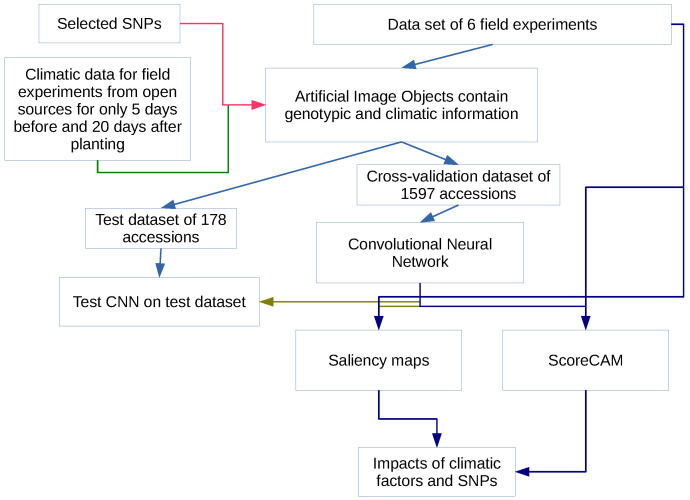
The overview of the research. The datasets, performed numerical experiments, and results are shown as boxes on the diagram, while the arrows represent the data flow.

The methodology proposed in this work consists of several steps (see Figure 1):

1. Identification of SNP with maximal influence on flowering time using three feature importance metrics for random forest models,
2. Construction of artificial images for each accession encoding information on the SNP values and climatic factors for a limited period of time,
3. Building a convolutional neural network for prediction of flowering time,
4. Investigation of the impacts of different factors on the model using two class activation mapping techniques.

These steps are described below.

### 3.2. Plant Material

The mung bean accessions from a WorldVeg collection described in [5] were phenotyped during several field experiments:

- 1984: sown on 28/08/1984; harvested on 24/10/1984, geographical coordinates: N 23${}^{\circ }$6${}^{\prime }$50${}^{\prime \prime }$ E 120${}^{\circ }$17${}^{\prime }$55${}^{\prime \prime }$;
- 1985: sown on 17/09/1985; harvested on 03/10/1985, geographical coordinates: N 23${}^{\circ }$6${}^{\prime }$50${}^{\prime \prime }$ E 120${}^{\circ }$17${}^{\prime }$55${}^{\prime }$;
- 2016: sown on 16/06/2016, harvested from 22/08 to mid-September, geographical coordinates: N 17${}^{\circ }$30${}^{\prime }$28${}^{\prime \prime }$ E 78${}^{\circ }$16${}^{\prime }$10${}^{\prime \prime }$;
- 2018: sown 21/09/2018 and harvested from 24–28 December 2018, geographical coordinates: N 23${}^{\circ }$6${}^{\prime }$50${}^{\prime \prime }$ E 120${}^{\circ }$17${}^{\prime }$55${}^{\prime \prime }$;
- 2018: sown 03/05/2018 and harvested from mid-July, geographical coordinates: N 45${}^{\circ }$18${}^{\prime }$ E 40${}^{\circ }$52${}^{\prime }$;
- 2019: sown 12/05/2018 and harvested from mid-July, geographical coordinates: N 46${}^{\circ }$14${}^{\prime }$ E 48${}^{\circ }$01${}^{\prime }$.

Details on the phenotyping experiments, genotyping, and subsequent analysis were presented in recent manuscripts. Genotype data were obtained using Diversity Arrays Technology Pty Ltd., Canberra, 353 Australia (DArTseq), and after further processing and filtering, 7916 SNPs were obtained [44,45].

### 3.3. SNP Selection

Machine learning methods, such as random forest [13], are an alternative to classical statistical approaches to the selection of markers associated with the phenotype. The main idea is to train the model to predict the flowering time for a given set of SNPs, and then use a Gini index, SHAP values, and the Boruta algorithm to find out which SNPs have the most significant impact on the prediction.

However, firstly the data are to be corrected for population structure. That is performed by multi-dimensional scaling (MDS). Next, the RF model is obtained and subjected to further analysis. To determine which SNPs were important and worthy of further study, importance scores were plotted, and a second-order inflection point [46] (“elbow method”) was determined. Predictors with importance values equal to or greater than the value at that point were identified as important SNPs [47].

#### 3.3.1. Accounting for Population Structure

To account for population structure in the dataset, we applied the method proposed in [48]. Briefly, given a genotype matrix (1)

(1) $$\begin{array}{c} X=\left(\begin{array}{cccc} x_{1,1} & x_{1,2} & \cdots & x_{1,m}\\ x_{2,1} & x_{2,2} & \cdots & x_{2,m}\\ \vdots & \vdots & \ddots & \vdots \\ x_{n,1} & x_{n,2} & \cdots & x_{n,m} \end{array}\right) \end{array}$$

where $x_{i,j}$ is the value of *j*-th SNP for the *i*-th accession. The similarity matrix $S=\{s_{i,k}\}$ is calculated using (2)

(2) $$s_{i,k}=\frac{1}{m}\sum_{j=1}^{m}I\left(x_{i,j},x_{k,j}\right),\;I\left(a,b\right)=\left\{\begin{array}{cc} 1, & a=b\\ 0, & a\neq b \end{array}\right.$$

Further multi-dimensional scaling (MDS) is used to reduce the number of dimensions. The matrix H is constructed (3)

(3) $$H=I^{n}-\frac{1}{n}J^{n}{\left(J^{n}\right)}^{T},\;J^{n}={\left(1,1,\cdots ,1\right)}^{T},$$

where $I^{n}$ is an identity matrix. Due to the symmetry of matrix *S*, matrix HSH is also symmetric. Consequently, *L* largest positive eigenvalues $\lambda_{1}\geq \lambda_{2}\geq \cdots \geq \lambda_{L}$ can be found together with corresponding eigenvectors $v_{p}$, p=1,⋯,L that define the directions with a large genetic variation.

Next, a k-medoids clustering algorithm [49] is applied to group accessions into separate clusters. The number of clusters *c* is determined so that for any $c^{*}>c$ the within-cluster dispersion is not sufficiently better.

Finally, the dataset is adjusted by subtracting the fitted dependence on the vector group membership [50].

#### 3.3.2. Random Forest

Detailed procedures of RF in a context of genetic association study have been described previously by [14]. Briefly, let $y\in R^{n}$ and $X\in R^{n\times m}$ denote the phenotype vector and genotype matrix, respectively.

To ‘grow’ a tree, RF begins by creating a bootstrap sample (with replacement) from the entire dataset. The remaining sample, which contains about one-third of the entire dataset, is called the ‘out-of-bag’ (OOB) sample [13].

A subset of SNPs, the size of which is the square root of *m* by default, is randomly selected at each node. The SNP with the greatest ability to improve the ‘purity’ of the child nodes is selected to split the node. The process of node splitting continues until the purity measurements of all terminal nodes cannot be improved. The procedure is repeated for *t* times to generate a forest with *t* trees.

For each tree in a forest, the outcome of each individual in the OOB sample can be predicted by letting the individual go down the tree.

After the entire forest is grown, an individual’s outcome would be determined as an average over all trees.

#### 3.3.3. Gini Index

Gini importance (or mean decrease impurity) is computed from the random forest structure. In the internal node of the individual tree, the selected feature is used to make decision how to divide the dataset into two separate sets. The features for internal nodes are selected with some criterion, which for classification tasks can be information gain and for regression is variance reduction [13]. Adding up the Gini improvement for each individual variable over all trees in the forest gives a fast variable importance [14].

#### 3.3.4. Boruta Algorithm

The Boruta algorithm [51] was specially developed as a powerful wrapper for the RF-based feature selection approach. The main principle of the Boruta algorithm is based on the extension of the attributes by adding random attributes to the dataset which are called shadow attributes and created by shuffling the original values of each attribute (in our case SNPs) in the dataset. The enlargement of the attributes results in apposition of the randomness to the dataset, which leads to the reduction of the bias of hidden (false) signals arising from random fluctuations or correlations in the dataset. To this end, an RF classifier is applied to the extended dataset, and those SNPs whose importance is significantly smaller than that of the shadow attributes are systematically and iteratively removed. By repeating the process of shadow attributes generation and RF algorithm application, importance is assigned to all SNPs. As a result, the Boruta algorithm provides a ranked list of SNPs with a decision of whether the importance of an SNP is confirmed, rejected, or tentative [16].

#### 3.3.5. SHAP Values

To overcome the drawbacks of model-specific interpretability more generalizable methods have been invented. One approach is to use Shapley values, which originated in game theory [15,52]. In Shapley values, each feature of the input data is treated as a player in a game where the outcome is the model’s prediction. Shapley values tell us how to fairly distribute the “payout” among the features [53]. Each player is removed from the dataset, and the average change in prediction outcome if the player (the data feature) is added to the game is calculated. The Shapley values are ordered numerically to infer a ranking of feature importance [54].

### 3.4. Climate Data

The data on daily values of climatic factors:

1. A day length *D*,
2. A minimal temperature $T_{n}$,
3. A maximal temperature $T_{x}$,
4. A precipitation *R*,
5. A relative humidity *H*,
6. A solar radiation *S*,

for field experiments were taken from the NASA Langley Research Center (LaRC) POWER Project funded through the NASA Earth Science/Applied Science Program [55].

### 3.5. Artificial Image Objects

Artificial image objects were used to encode information on $V_{g}$ genotypic and $V_{c}$ climatic features for each accession. The information on weather conditions for only 5 days before and 20 days after planting was used as input to the model. Thus, the number of climatic features $V_{c}=25\times 6=150$. The total number of features that equals the number of pixels in an AIO was $V_{t}=V_{g}+V_{c}$.

While the dimensions of AIOs may be selected arbitrarily, it was decided to set the number of rows equal to the number of climatic factors, namely 6, and the number of columns varied according to the number of selected SNPs *K*. AIO I(x,y) can be represented as a matrix (4) with two blocks $I_{g}$ and $I_{c}$ for genotypic and climatic data, respectively.

(4) $$\begin{array}{c} I\left(x,y\right)=\left[\begin{array}{ccc} I_{g}\left(x,y\right) & | & I_{c}\left(x,y\right) \end{array}\right]\\ =\left[\begin{array}{cccccc} i_{g}\left(1,1\right) & i_{g}\left(1,2\right)\cdots & i_{g}\left(1,\frac{K}{6}\right) & i_{c}\left(1,\frac{K}{6}+1\right) & i_{c}\left(1,\frac{K}{6}+2\right)\cdots & i_{c}\left(1,\frac{K}{6}+25\right)\\ i_{g}\left(2,1\right) & i_{g}\left(2,2\right)\cdots & i_{g}\left(2,\frac{K}{6}\right) & i_{c}\left(2,\frac{K}{6}+1\right) & i_{c}\left(2,\frac{K}{6}+2\right)\cdots & i_{c}\left(2,\frac{K}{6}+25\right)\\ \vdots & \vdots & \vdots & \vdots & \vdots & \vdots \\ i_{g}\left(6,1\right) & i_{g}\left(6,2\right)\cdots & i_{g}\left(6,\frac{K}{6}\right) & i_{c}\left(6,\frac{K}{6}+1\right) & i_{c}\left(6,\frac{K}{6}+2\right)\cdots & i_{c}\left(6,\frac{K}{6}+25\right) \end{array}\right] \end{array}$$

Each pixel value $i_{g}$ or $i_{c}$ combines three channels, *R*, *G*, and *B*, for three pseudo colors, red, green and blue, respectively.

The value of climatic factor $f_{c}$ was converted to a pixel value $i_{c}\left(x,\frac{K}{6}+y\right)$ according to (5).

(5) $$G=\left\{\begin{array}{cc} 0, & f_{c}>0\\ 1, & f_{c}\leq 0 \end{array}\right.\;R=f_{c}\;\mathrm{div}\;255,\;B=f_{c}\;\mathrm{mod}\;255,$$

where *x* is defined by the number of factors in the enumeration in Section 3.4, and *y* is the number of the day starting from the fourth day before planting.

The value $f_{g}$ of SNP with index *k* was converted to a pixel value $i_{g}\left(x,y\right)$ according to (6).

(6) $$R=\left\{\begin{array}{cc} 1, & f_{g}=0\\ 0, & f_{g}\neq 0 \end{array}\right.\;G=\left\{\begin{array}{cc} 1, & f_{g}=1\\ 0, & f_{g}\neq 1 \end{array}\right.\;B=\left\{\begin{array}{cc} 1, & f_{g}=2\\ 0, & f_{g}\neq 2 \end{array}\right.$$

where (x−5)×y=k.

### 3.6. Convolutional Neural Network

The model for flowering time was built in the form of a convolutional neural network that takes artificial image objects as input [18,19] and predicts a class that corresponds to the time to flowering in days. Since we are working with three-channel color images in this study, each filter is a collection of three kernels. Each kernel slides along the corresponding image channel; the result of processing the kernels is combined into one feature map. The size of the filter kernel of each convolutional layer (Conv2D type) was found by adapting to the available experimental data. The values of the weights of the convolutional kernels are the learning parameters of the neural network. Each convolutional layer is followed by a subsampling layer (max pooling 2D type), the purpose of which is to reduce the dimension of maps in order to enlarge features. Such filtering helps, among other things, to avoid overfitting. The formation of a new feature map is based on the max pooling operation, which is performed by selecting the maximum value from a subsample of a given size. The number of feature maps in the output remains unchanged. At the last stage, each feature map is expanded into a vector (flatten type layer); the resulting vectors are concatenated into a single numerical series, which is fed to the input of a fully connected neural network (dense type). The task of the network is to determine the probability with which the input image belongs to each class. The number of neurons in the output layer corresponds to the number of recognized classes.

In this study, we used the TensorFlow and Keras to optimize the architecture and the weights of CNN.

The categorical cross-entropy was chosen as the loss function for the convolutional neural network. The purpose of this cost function is to measure the distance of the output probabilities $T_{i}$ from the true values $S_{i}$ (7).

(7) $$L_{CE}\left(S,T\right)=-\sum_{i=1}T_{i}\log\left(S_{i}\right)$$

The accuracy metric shows the proportion of correctly affixed class labels (8).

(8) $$a=\frac{TP+TN}{TP+TN+FP+FN}$$

where TP and TN are the number of true positive and negative decisions, respectively, and FP and FN are false positive and false negative ones, respectively.

### 3.7. Impacts of Different Factors to the Model Solution

Among the available approaches, two visual explanation methods, namely saliency map [23] and Score-CAM [20], were used to evaluate the impacts of genotypic and climatic factors to the model solution.

The saliency map visualizes which pixels of the image contribute the most to the prediction [23]. Score-CAM was developed as a novel post hoc visual explanation method based on class activation mapping. Unlike previous approaches, Score-CAM eliminates the dependence on gradients by obtaining the weight of each activation map through its forward passing score on target class. The final result is obtained by a linear combination of weights and activation maps [20].

Both types of maps were computed for each individual AIO, i.e., for each accession, and averaged over accessions belonging to particular class. Thus, the most important factors and their combinations can be determined visually by comparing these maps with the structure of the AIO.

## 4. Results

### 4.1. Selected SNPs

The available dataset was corrected to account for population structure using an MDS algorithm that recovered five subpopulations. The corrected data were used to fit a series of random forest model hyperparameters, such as “max_depth”, “min_samples_split”, “n_estimators”, “min_impurity_decrease”, and “max_features” which were optimized by Bayesian optimization using a Gaussian processes method from the skopt package for Python. Feature importances were then computed using a Gini index, SHAP values, and the Boruta algorithm, and thresholds were selected by a second-order inflection point (“elbow method”).

**Figure 2:**
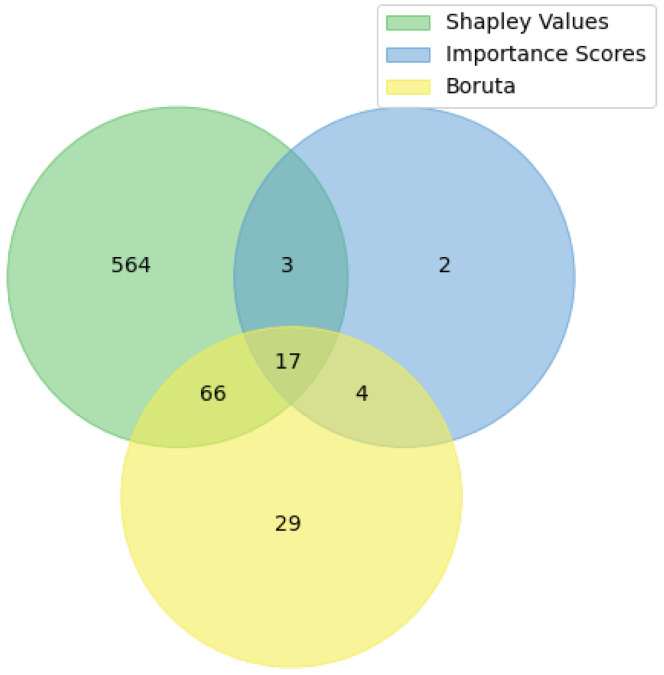
The Venn diagram for the selected SNP. The selection was performed using importance scores, the Boruta algorithm, and SHAP values.

The application of three methods resulted in different sets of high-ranked features that intersect to some extent (see Figure 2). Consequently, two sets of SNPs were obtained using the proposed methodology:

- The cross-SNP set includes 17 SNPs from the intersection of all three results (see Table 1),
- The union SNP set includes 90 SNPs from pair-wise intersections of three results (see Zenodo link in Data Availability Statement).

### 4.2. Model for Time to Flowering

**Figure 3:**
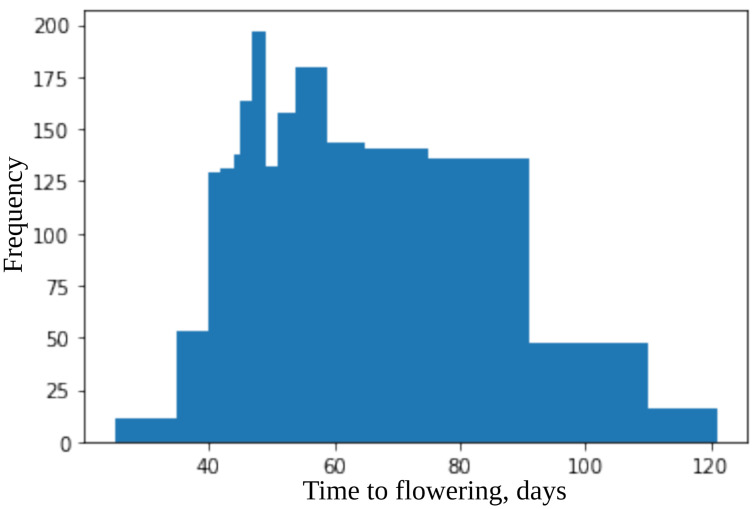
Histogram of time to flowering for dataset.

The time to flowering ranges from 25 to 120 days (see Figure 3). The whole set of accessions was subdivided into 15 classes with maximal time to flowering: 35, 40, 42, 44, 45, 47, 49, 51, 54, 59, 65, 75, 91, 110, and 120. The number of accessions in these classes were: 11, 53, 129, 131, 138, 163, 197, 132, 158, 180, 143, 141, 136, 47, and 16.

**Figure 4:**
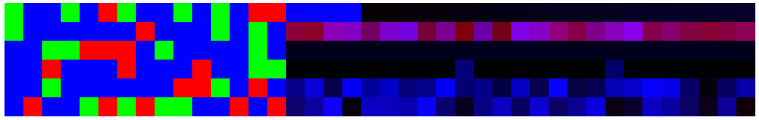
Example AIO for the union SNP set. The data on SNP and climate occupies the left and right sides of the image, respectively. The size of the image is 6 × 40 px. Here, the image is enlarged; each colored square corresponds to one pixel. The color of each pixel is obtained by (6) and (5) for genotypic and climatic factors, respectively.

**Figure 5:**
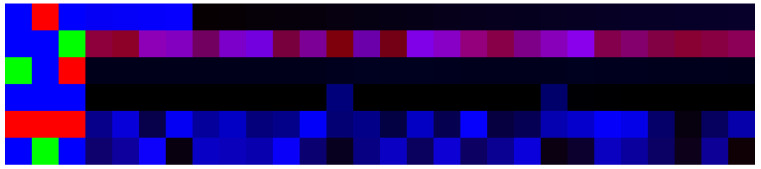
Example AIO for the cross-SNP set for the same accession as in Figure 4. The data on SNP and climate occupies the left and right sides of the image, respectively. The size of the image is 6 × 28 px. Here, the image is enlarged; each colored square corresponds to one pixel. The color of each pixel is obtained by (6) and (5) for genotypic and climatic factors, respectively.

The artificial image objects were constructed for all accessions and both the union and cross-SNP sets. AIOs provide a convenient visualization for the data (see Figure 4 and Figure 5). The colors of pixels in the AIOs are defined by Equations (5) and (6) for genotypic and climatic factors, respectively.

The CNN was trained to classify artificial images according to measured flowering time using 1597 accessions for cross-validation and 178 for control (see Figure 1). As a result of applying the convolution operation on each convolution layer, the pixel values of the corresponding fragment of the input image are multiplied element by element by the convolution kernel. The result is summarized and written to a certain position of the output image, which is called feature maps. Thus, the input image patterns are analyzed using filters, each of which is responsible for extracting one specific feature.

To achieve the best performance of the model, it is necessary to solve the problem of optimizing the hyperparameters of the training algorithm. Hypertuning is a time-consuming process that is often performed manually and is computationally intensive. In order to effectively solve the problem of tuning the hyperparameters of a convolutional neural network, such as the number of filters of each layer, the activation function, and the presence of a batch normalization layer, the Keras Tuner library was used. Keras Tuner is a deep learning library that is a high-level API in the Python programming language based on TensorFlow.

When building the hypertuning model, a tuner instance was created that solves the optimization problem by examining the range of values defined for each of the hyperparameters in addition to the architecture of the model. All hyperparameters of the model constructed in this study are discrete. The model builder function iterates over the parameters of the given space and returns the best model that meets the specified quality criteria.

For the union SNP set using the keras_tuner package, the model with 7 layers was obtained with 28,065 trainable parameters (see Table 2).

**Figure 6:**
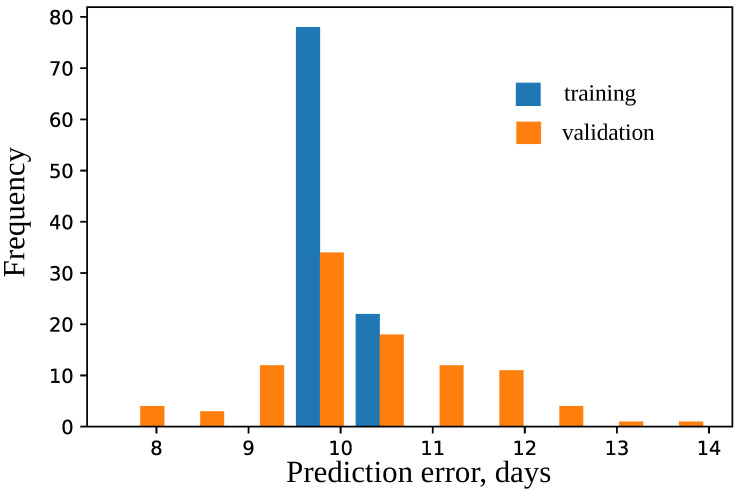
Histogram of errors in days for training and validation sets for models for the union SNP set. Mean values are 10.105 and 10.2575, respectively.

**Figure 7:**
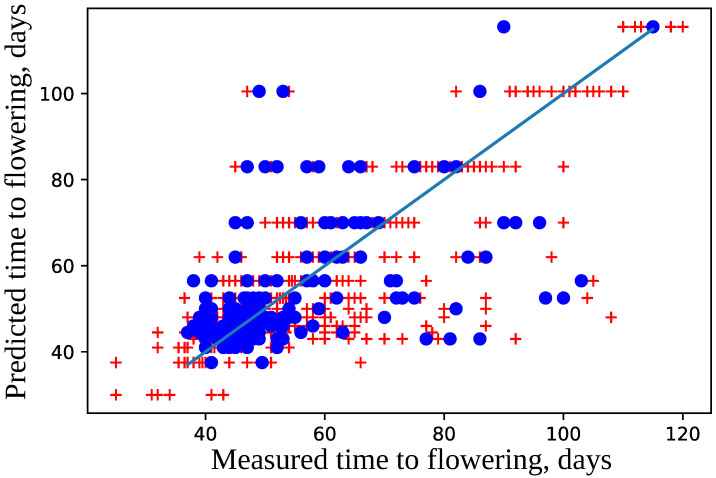
Comparison of measured and predicted flowering time. The data points used for training are marked with red crosses, and those from the test set are drawn as blue dots. The straight line represents the exact correspondence. The model accuracy was a=85%.

The model was trained using 10-fold cross-validation and parameters epochs = 100, and validation_split = 0.2. The difference in prediction errors on the training and validation data was statistically insignificant according to Mann–Whitney criterion with U = 4609.5 and P = 0.34 and Wilcoxon criterion W = 1232.0 and P = 0.23 (see Figure 6). The best model was selected that had the maximal accuracy for the validation set. The best model predicts flowering time for the test dataset with high accuracy (see Figure 7); the median error is 5 days.

For the cross-SNP set using the keras_tuner package, the model with 7 layers was obtained with 28,065 trainable parameters (see Table 3).

**Figure 8:**
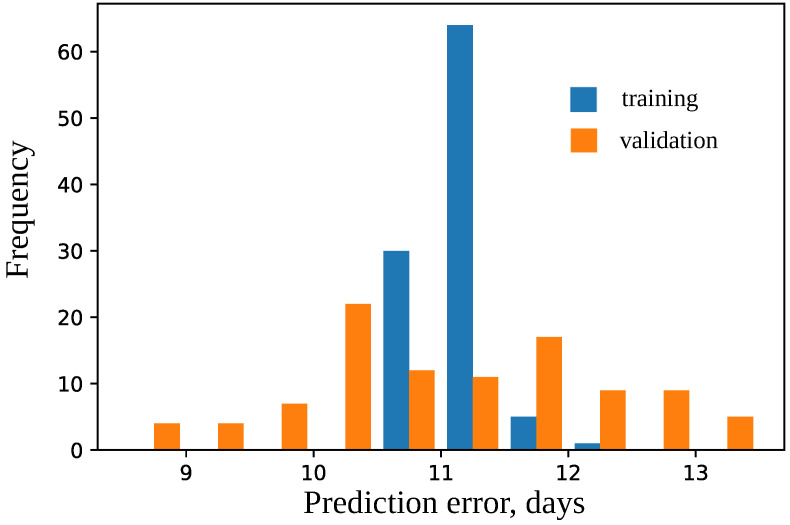
Histogram of errors in days for training and validation sets for models for the union SNP set. Mean values are 10.893 and 10.905, respectively.

**Figure 9:**
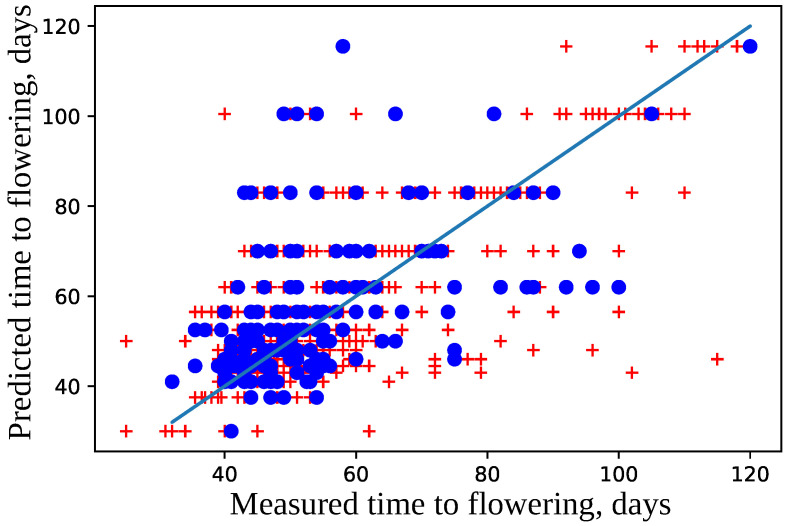
Comparison of measured and predicted flowering time. The data points used for training are marked with red crosses, and those from the test set are drawn as blue dots. The straight line represents the exact correspondence. The model accuracy is a=79%.

The model was trained using 10-fold cross-validation; parameters epochs = 100, and validation_split = 0.2. The difference in prediction errors on the training and validation data was statistically insignificant according to Mann–Whitney criterion with U = 5053.0 and P = 0.90 and Wilcoxon criterion W = 2074.0 and P = 0.94 (see Figure 8). The best model was selected that had the maximal accuracy for the validation set. The best model predicts flowering time for the test dataset with high accuracy (see Figure 9); the median error is 6.5 days.

### 4.3. Important Features for Models Based on the Cross-SNP Set

**Figure 10:**
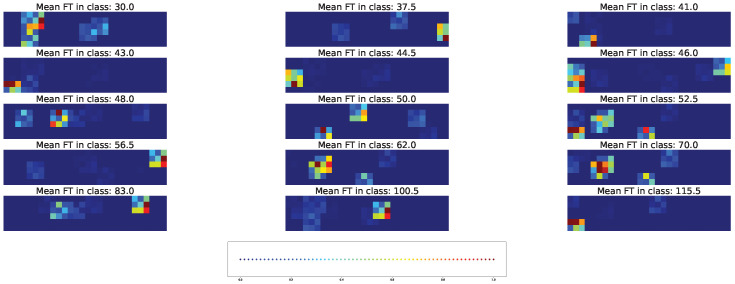
Example of the saliency map for individual acccessions in each time class and cross-SNP set. Figure S1 gives the same data with higher magnification.

**Figure 11:**
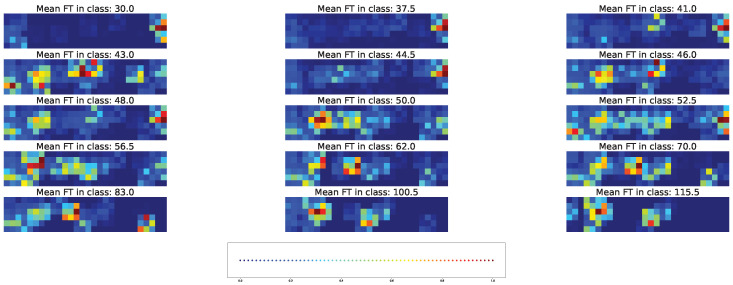
Average saliency maps for time classes and cross-SNP set. Figure S2 gives the same data with higher magnification.

**Figure 12:**
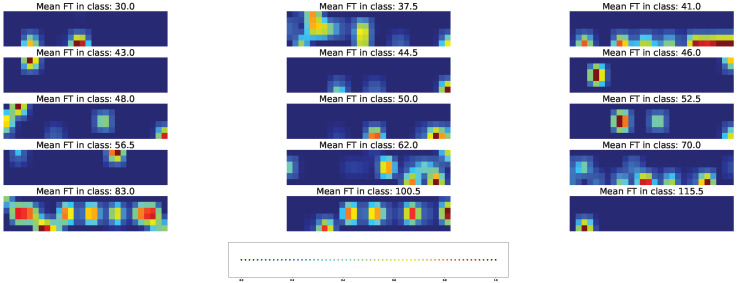
Example of the Score-CAM map for individual acccessions in each time class and cross-SNP set. Figure S3 gives the same data with higher magnification.

**Figure 13:**
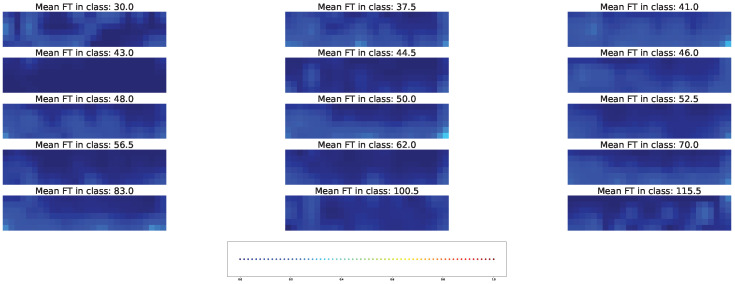
Average Score-CAM maps for time classes and cross-SNP set. Figure S4 gives the same data with higher magnification.

Two types of visual explanation methods, namely saliency map [23] and Score-CAM [20], were applied to the resulting models in order to determine the most important genotypic and climatic factors. The saliency maps (see Figure 10 and Figure 11) and Score-CAM maps (see Figure 12 and Figure 13) were computed for each individual accession and averaged over accessions belonging to a particular class for the cross-SNP set.

For the models based on the cross-SNP set, the highly activated pixels in the average saliency maps were distributed rather homogeneously within the figure for a fixed time class, indicating that both SNPs (three leftmost columns of pixels in the figures) and climatic factors (all other columns) essentially participate in determining the time to flowering (Figure 11). Considering how the activation patterns in these average saliency maps change with the flowering time class, we can see that a higher number of SNPs and climatic factors are involved for the middle times (43–70 days), and this number is smaller for early and late times. The actual set of these factors stay approximately the same for all times but vary their importance levels (pixel brightness in the figure) with time.

A visible distinction between the individual and averaged saliency maps (Figure 11 vs. Figure 10) indicates that individual accessions may significantly deviate from the average picture in terms of the number of important features. The individual activation patterns also demonstrate higher variability across the flowering time classes. On the other hand, the fact that the average picture contains bright pixel clusters means that the averaging does not flatten the individual saliency maps, which stems from the fact that the majority of accessions share the same set of important SNPs and climatic factors.

Average saliency maps highlighted the importance of SNPs: “Vr8, position 41054773”, “Vr7, position 50907346”, and “Vr8, position 41054763”. Humidity on different days before and after planting was marked to have high importance for model prediction for all classes. Minimal temperature was highlighted for classes with mean FT 30, 37, 41, 50, 52, 100, and 115 days, while maximal temperature was a marker for classes with mean FT of 43 and 83 days.

Important feature selection based on the Score-CAM maps predicts a different type of individual variability between the features. Individual accessions may exhibit a pattern of important genetic and climatic factors distributed across the time classes with a density that is visually similar to the case of the saliency maps (Figure 12). However, these patterns are averaged to almost no activation (Figure 13), which means that different accessions possess different important factors according to the Score-CAM method.

### 4.4. Important Features for Models Based on the Union SNP Set

**Figure 14:**
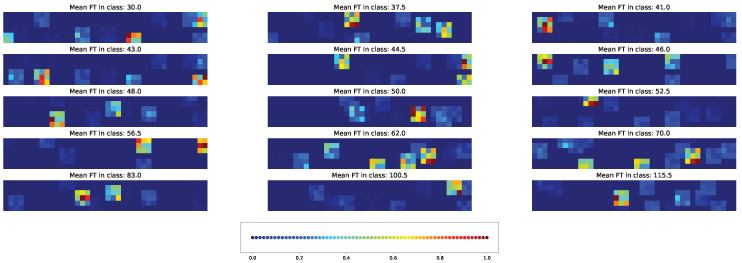
Example of the saliency map for individual acccessions in each time class and union SNP set. Figure S5 gives the same data with higher magnification.

**Figure 15:**
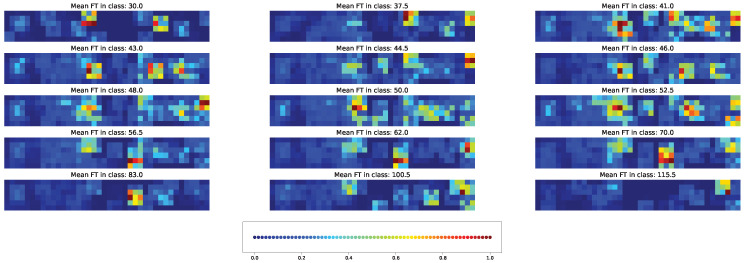
Average saliency maps for time classes and the union SNP set. Figure S6 gives the same data with higher magnification.

**Figure 16:**
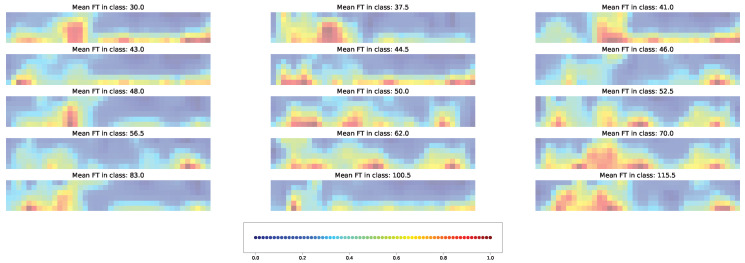
Example of the Score-CAM map for individual acccessions in each time class and union SNP set. Figure S7 gives the same data with higher magnification.

**Figure 17:**
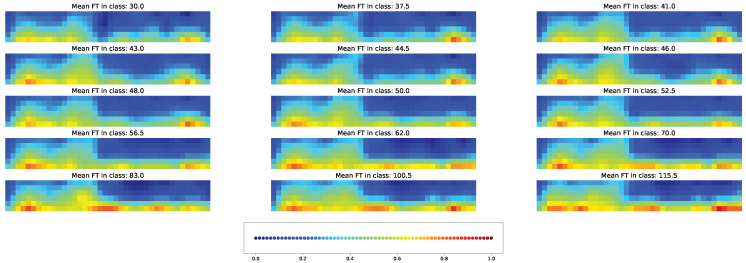
Average Score-CAM maps for time classes and the union SNP set. Figure S8 gives the same data with higher magnification.

Both types of visual explanation methods were applied to the resulting models for the union SNP set. The individual and averaged saliency maps (Figure 14 and Figure 15) and Score-CAM maps (Figure 16 and Figure 17) were computed similarly to the cross-SNP set.

Average saliency maps highlighted the importance of SNPs “Vr5, pos. 4627938” and “Vr1, pos.13512065” for classes with mean FT of 30, 43, 48, 50, 52, 56, and 83 days. Among climatic factors, humidity and precipitation were marked as important for all classes at some days after planting and also before planting for classes with mean FT of 43 and 50 days. Minimal temperature in the beginning or in the middle of the considered time interval showed importance for several classes, while the maximal temperature and solar radiation on days 19 and 29 were important for only some of them.

Average Score-CAM maps highlighted for all clasess the importance of seven SNPs: “Vr9, pos.1712557”, “Vr1, pos.33528594”, “Vr8, pos.41054763”, “Vr7, pos.31361558”, and “Vr7, pos.50907346”. SNPs “Vr7, pos.50907346” and “Vr8, pos.41054763” were also highlighted for the cross-SNP set. According to this approach, the main climatic factor was maximal temperature that gained the highest importance on periods from 3 days before to 4 days after planting and days 15–20 after planting.

As in the case of the cross-SNP set, both individual and averaged saliency maps for the models based on the union SNP set demonstrate the involvement of both genetic and climatic factors in predicting time to flowering (Figure 14 and Figure 15). In this case, however, the brightness of the averaged saliency maps are visibly shifted to climatic factors. This indicates that, according to the saliency maps, accessions share common climatic factors as important features but differ significantly in terms of important SNPs.

We observe a reverse tendency when important features are selected via the Score-CAM maps. The averaged maps highlight SNPs as more important than climatic factors (Figure 17). All important features change only slightly with the time class. Individual Score-CAM maps may demonstrate a higher minimal brightness (Figure 16), indicating that each factor in the model has a nonvanishing input in flowering time prediction. Overall, the activation patterns on the Score-CAM maps significantly differ from those on the saliency maps for the models using the union SNP set.

## 5. Discussion

Mung bean (*Vigna radiata* (L.) Wilczek) is used in several traditional cuisines across Asia as a rich source of proteins and micronutrients. It has been an orphan tropical crop for a long time but could fulfill a role in a range of agroecologies. It fits into crop rotations due to a short duration cycle. One setting where mung bean fits well is in rotation with winter wheat, which is often harvested late in spring, leaving only a short summer season for mung bean; short duration is particularly critical to set seed before cool weather begins in this role [29]. Development of new varieties adapted to different conditions is necessary to meet the needs of growing global demand. The adaptation of adaptive traits such as flowering time to specific environments is blueprinted in genomes [56,57] so that different genotypes respond to local conditions in different ways. Though the amount of accumulated data is constantly growing, the understanding of the role of temperature and day length in adaptation to different agroecological conditions is still incomplete [58].

Here, we proposed a new modeling approach in which the data on genotypic and climatic factors for each accession were encoded as an artificial image object and used to train a convolutional neural network that predicted time to flowering. The important SNPs were identified using random forest and three feature importance techniques. The dataset consisted of 1775 accessions phenotyped in six different environments. The climatic factors included daily values of maximal and minimal temperature, precipitation, day length, and solar radiation.

To illustrate the methodology, two sets of SNPs were used to build the models. The cross-SNP set consisted of 17 markers identified by all three feature selection methods, and the union SNP set included 90 markers identified by at least two algorithms. Both CNN models predicted flowering time with high accuracy in which the median error was 5 and 7 days for the union and cross-SNP sets, respectively. In contrast to previous modeling attempts [28,29], the presented approach uses only limited information on daily weather, namely 5 days before and 20 days after planting, that may make it possible to predict the day of flowering in real life.

The impacts of specific factors to the model solution were analysed by visual explanation methods, namely a saliency map and Score-CAM. The results showed that different values of time to flowering are determined by different genotypic and climatic factors.

We showed that the saliency and Score-CAM maps can be useful for visual representation of both genetic and climatic factors that are important features in predicting time to flower. However, the two feature selection methods may lead to different qualitative conclusions depending on the context in which they are used. When the smaller list of SNPs was used in the model, the two methods demonstrated different types of variation of important features across individual accessions. For the larger list of SNPs, these methods assigned different importance to genetic and climatic factors, with climatic factors highlighted as more important on the saliency maps and SNPs on the Score-CAM maps. Our results indicate that multiple methods should be used as a control for any predictions about which factors are important or not.

The SNPs “Vr8, position 41054773” and “Vr8, position 41054763” are located in exon Vradi08g19140.1. The gene is associated with “embryo development ending in seed dormancy” biological process according to PLAZA 5.0 [59].

As CNN-based models deal with the features extracted from images, it is important what part of the AIO is reserved for either genetic or climatic factors. The cross-SNP set contains SNPs that have a higher potential to be important, but at the same time, this set occupies less fraction of the AIO and, hence, might be eventually transformed into feature maps with less information content compared to the fixed set of climatic factors. The difference in importance that the saliency and Score-CAM maps assign to the two types of factors in the model can be related to the fact that these methods deal differently with the fractions of information contained in the AIO. This may indicate that the SNP selection problem has an additional aspect in the context of using these SNPs in models based on AIO analysis, as this selection should be additionally considered as a trade-off between the significance level analysis and the relative size of the visual information that the selected SNPs represent in the AIO.

## 6. Conclusions

The proposed methodology is capable of identifying important SNPs and efficiently encoding genotypic and climatic factors as AIOs. The proposed CNN uses weather data for only the limited time period of 5 days before and 20 days after planting and is capable of predicting time to flowering of accessions from a mung bean dataset with high accuracy. The most important factors that influence the model solution were identified using two techniques. Future research in the application of artificial image objects and machine learning methods to identify important SNP markers and construct predictive models of important agronomic traits will include optimization of the layout of factors in AIOs and model verification with independent datasets.

## Figures and Tables

**Table 1:** The 17 Selected SNPs that make up the cross-SNP set.

Num	Chr	Pos	Major	Minor
0	1	5205070	G	A
1	8	41054773	A	G
2	1	35363026	C	T
3	11	13512065	T	C
4	8	14888418	C	T
5	1	33528594	A	T
6	scaffold 375	174056	T	G
7	7	50907346	G	A
8	scaffold 209	359166	G	A
9	9	1712557	A	G
10	6	15592645	T	C
11	scaffold 261	18461	A	G
12	3	2134396	A	G
13	8	41054763	A	G
14	scaffold 343	148518	A	G
15	scaffold 207	213949	A	G
16	8	1049804	A	G

**Table 2:** Model architecture for the union SNP set. The types and names of the layers of constructed CNN are given in the first column, while the numbers of outgoing connections and the number of trainable parameters are in the second and the last column, respectively (see details in Section 3.6).

Layer (Type)	Output Shape	Param Number
conv2d (Conv2D)	(6, 40, 10)	280
max_pooling2d (MaxPooling2D)	(3, 20, 10)	0
conv2d_1 (Conv2D)	(3, 20, 50)	4550
max_pooling2d_1 (MaxPooling2D)	(1, 10, 50)	0
flatten (Flatten)	(500)	0
dense (Dense)	(45)	22,545
dense_1 (Dense)	(15)	690

**Table 3:** Model architecture for the cross-SNP set.The types and names of the layers of the constructed CNN are given in the first column, while the number of outgoing connections and the number of trainable parameters are in the second and the last column, respectively (see details in Section 3.6).

Layer (Type)	Output Shape	Param Number
conv2d (Conv2D)	(6, 28, 7)	196
max_pooling2d (MaxPooling2D)	(3, 14, 7)	0
conv2d_1 (Conv2D)	(3, 14, 42)	2688
max_pooling2d_1 (MaxPooling2D)	(1, 7, 42)	0
flatten (Flatten)	(294)	0
dense (Dense)	(45)	13,275
dense_1 (Dense)	(15)	690

## Data Availability

The data analyzed in this study are available in Zenodo at 10.5281/zenodo.7376218.

## Supplementary Materials

The following are available online at https://www.mdpi.com/article/10.3390/plants11233327/s1, Figure S1: The saliency map for individual acccessions in each time class and cross-SNP set, Figure S2: Average saliency maps for time classes and cross-SNP set, Figure S3: The Score-CAM map for individual acccessions in each time class and cross-SNP set, Figure S4: Average Score-CAM maps for time classes and cross-SNP set, Figure S5: The saliency map for individual acccessions in each time class and union SNP set, Figure S6: Average saliency maps for time classes and union SNP set, Figure S7: The Score-CAM map for individual acccessions in each time class and union SNP set, Figure S8: Average Score-CAM maps for time classes and union SNP set.

## References

[1] Chivenge P., Mabhaudhi T., Modi A., Mafongoya P. (2015). The Potential Role of Neglected and Underutilised Crop Species as Future Crops under Water Scarce Conditions in Sub-Saharan Africa. Int. J. Environ. Res. Public Health.

[2] Vara-Ubol S., Chambers E., Chambers D.H. (2004). Sensory characteristics of chemical compounds potentially associated with beany aroma in foods. J. Sens. Stud..

[3] Vishnyakova M.A., Burlyaeva M.O., Samsonova M.G. (2018). Green gram and black gram: Prospects of cultivation and breeding in Russian Federation. Vavilov J. Genet. Breed..

[4] Burlyaeva M., Vishnyakova M., Gurkina M., Kozlov K., Lee C.R., Ting C.T., Schafleitner R., Nuzhdin S., Samsonova M., von Wettberg E. (2019). Collections of Mungbean [*Vigna radiata* (L.) R. Wilczek] and urdbean [*V. mungo* (L.) Hepper] in Vavilov Institute (VIR): Traits diversity and trends in the breeding process over the last 100 years. Genet. Resour. Crop. Evol..

[5] Schafleitner R., Nair R.M., Rathore A., Wang Y.W., Lin C.Y., Chu S.H., Lin P.Y., Chang J.C., Ebert A.W. (2015). The AVRDC—The World Vegetable Center mung bean (*Vigna radiata*) core and mini core collections. BMC Genom..

[6] Singh V., Yadav N.R., Singh J. (2017). Role of Genomic tools for Mungbean [*Vigna radiata* (L.) Wilczek] improvement. Legume Res. Int. J..

[7] Kang Y.J., Kim S.K., Kim M.Y., Lestari P., Kim K.H., Ha B.K., Jun T.H., Hwang W.J., Lee T., Lee J. (2014). Genome sequence of mung bean and insights into evolution within Vigna species. Nat. Commun..

[8] Kim S.K., Nair R.M., Lee J., Lee S.H. (2015). Genomic resources in mung bean for future breeding programs. Front. Plant Sci..

[9] Swindell R., Poehlman J.M. (1978). Inheritance of photoperiod response (*Vigna radiata* [L.] wilczek). Euphytica.

[10] Ellis R.H., Lawn R.J., Summerfield R.J., Qi A., Roberts E.H., Chay P.M., Brouwer J.B., Rose J.L., Yeates S.J., Sandover S. (1994). Towards the Reliable Prediction of Time to Flowering in Six Annual Crops. IV. Cultivated and Wild Mung Bean. Exp. Agric..

[11] Imrie B.C., Drake D.W., Delacy I.H., Byth D.E. (1981). Analysis of genotypic and environmental variation in international mung bean trials. Euphytica.

[12] Nath D., Dasgupta T. (2013). Genotype × Environment Interaction and Stability Analysis in Mungbean. IOSR J. Agric. Vet. Sci..

[13] Breiman L. (2001). Random Forests. Mach. Learn..

[14] Sun Y.V. (2010). Multigenic Modeling of Complex Disease by Random Forests. Advances in Genetics.

[15] Shapley L.S., Roth A.E. (1988). The Shapley Value: Essays in Honor of Lloyd S. Shapley.

[16] Ramzan F., Klees S., Schmitt A.O., Cavero D., Gültas M. (2020). Identification of Age-Specific and Common Key Regulatory Mechanisms Governing Eggshell Strength in Chicken Using Random Forests. Genes.

[17] Piekutowska M., Niedbała G., Piskier T., Lenartowicz T., Pilarski K., Wojciechowski T., Pilarska A.A., Czechowska-Kosacka A. (2021). The Application of Multiple Linear Regression and Artificial Neural Network Models for Yield Prediction of Very Early Potato Cultivars before Harvest. Agronomy.

[18] Chen X., Chen D.G., Zhao Z., Zhan J., Ji C., Chen J. (2021). Artificial image objects for classification of schizophrenia with GWAS-selected SNVs and convolutional neural network. Patterns.

[19] Chen X., Chen D.G., Zhao Z., Balko J.M., Chen J. (2021). Artificial image objects for classification of breast cancer biomarkers with transcriptome sequencing data and convolutional neural network algorithms. Breast Cancer Res..

[20] Wang H., Wang Z., Du M., Yang F., Zhang Z., Ding S., Mardziel P., Hu X. Score-CAM: Score-Weighted Visual Explanations for Convolutional Neural Networks. Proceedings of the 2020 IEEE/CVF Conference on Computer Vision and Pattern Recognition Workshops (CVPRW).

[21] Zhang X., Gao J. (2020). Measuring Feature Importance of Convolutional Neural Networks. IEEE Access.

[22] Selvaraju R.R., Cogswell M., Das A., Vedantam R., Parikh D., Batra D. (2020). Grad-CAM: Visual Explanations from Deep Networks via Gradient-Based Localization. Int. J. Comput. Vis..

[23] Simonyan K., Vedaldi A., Zisserman A. (2014). Deep Inside Convolutional Networks: Visualising Image Classification Models and Saliency Maps. arXiv.

[24] Selvaraju R.R., Cogswell M., Das A., Vedantam R., Parikh D., Batra D. Grad-CAM: Visual Explanations from Deep Networks via Gradient-Based Localization. Proceedings of the 2017 IEEE International Conference on Computer Vision (ICCV).

[25] Ageev A., Aydogan A., Bishop-von Wettberg E., Nuzhdin S.V., Samsonova M., Kozlov K. (2021). Simulation Model for Time to Flowering with Climatic and Genetic Inputs for Wild Chickpea. Agronomy.

[26] Ageev A.Y., Bishop-von Wettberg E.J., Nuzhdin S.V., Samsonova M.G., Kozlov K.N. (2021). Forecasting the Timing of Floral Initiation in Wild Chickpeas under Climate Change. Biophysics.

[27] Kozlov K., Singh A., Berger J., Wettberg E.B.V., Kahraman A., Aydogan A., Cook D., Nuzhdin S., Samsonova M. (2019). Non-linear regression models for time to flowering in wild chickpea combine genetic and climatic factors. BMC Plant Biol..

[28] Ageev A., Lee C.R., Ting C.T., Schafleitner R., Bishop-von Wettberg E., Nuzhdin S.V., Samsonova M., Kozlov K. (2021). Modeling of Flowering Time in Vigna radiata with Approximate Bayesian Computation. Agronomy.

[29] Kozlov K., Sokolkova A., Lee C.R., Ting C.T., Schafleitner R., Bishop-von Wettberg E., Nuzhdin S., Samsonova M. (2020). Dynamical climatic model for time to flowering in Vigna radiata. BMC Plant Biol..

[30] Boote K.J., Jones J., Pickering N. (1996). Potential Uses and Limitations of Crop Models. Agron. J..

[31] Jones J., Hoogenboom G., Porter C., Boote K., Batchelor W., Hunt L., Wilkens P., Singh U., Gijsman A., Ritchie J. (2003). The DSSAT cropping system model. Eur. J. Agron..

[32] Mabhaudhi T., Chibarabada T.P., Chimonyo V.G.P., Modi A.T. (2018). Modelling climate change impact: A case of bambara groundnut (Vigna subterranea). Phys. Chem. Earth Parts A/B/C.

[33] Chapman S.C., Cooper M., Hammer G.L., Butler D.G. (2000). Genotype by environment interactions affecting grain sorghum. II. Frequencies of different seasonal patterns of drought stress are related to location effects on hybrid yields. Aust. J. Agric. Res..

[34] Soltani A., Khooie F., Ghassemi-Golezani K., Moghaddam M. (2001). A simulation study of chickpea crop response to limited irrigation in a semiarid environment. Agric. Water Manag..

[35] Chauhan Y.S., Douglas C., Rachaputi R.C.N., Agius P., Martin W., Skerman A. Physiology of mung bean and development of the mung bean crop model. Proceedings of the 1st Australian Summer Grains Conference.

[36] Mieth B., Kloft M., Rodríguez J.A., Sonnenburg S., Vobruba R., Morcillo-Suárez C., Farré X., Marigorta U.M., Fehr E., Dickhaus T. (2016). Combining Multiple Hypothesis Testing with Machine Learning Increases the Statistical Power of Genome-wide Association Studies. Sci. Rep..

[37] Paré G., Mao S., Deng W.Q. (2017). A machine-learning heuristic to improve gene score prediction of polygenic traits. Sci. Rep..

[38] Nicholls H.L., John C.R., Watson D.S., Munroe P.B., Barnes M.R., Cabrera C.P. (2020). Reaching the End-Game for GWAS: Machine Learning Approaches for the Prioritization of Complex Disease Loci. Front. Genet..

[39] Nguyen T.T., Huang J.Z., Wu Q., Nguyen T.T., Li M.J. (2015). Genome-wide association data classification and SNPs selection using two-stage quality-based Random Forests. BMC Genom..

[40] Szymczak S., Holzinger E., Dasgupta A., Malley J.D., Molloy A.M., Mills J.L., Brody L.C., Stambolian D., Bailey-Wilson J.E. (2016). r2VIM: A new variable selection method for random forests in genome-wide association studies. BioData Min..

[41] Stephan J., Stegle O., Beyer A. (2015). A random forest approach to capture genetic effects in the presence of population structure. Nat. Commun..

[42] Chattopadhay A., Sarkar A., Howlader P., Balasubramanian V.N. Grad-CAM++: Generalized Gradient-Based Visual Explanations for Deep Convolutional Networks. Proceedings of the 2018 IEEE Winter Conference on Applications of Computer Vision (WACV).

[43] Jiang P.T., Zhang C.B., Hou Q., Cheng M.M., Wei Y. (2021). LayerCAM: Exploring Hierarchical Class Activation Maps for Localization. IEEE Trans. Image Process..

[44] Sokolkova A., Burlyaeva M., Valiannikova T., Vishnyakova M., Schafleitner R., Lee C.R., Ting C.T., Nair R.M., Nuzhdin S., Samsonova M. (2020). Genome-wide association study in accessions of the mini-core collection of mung bean (Vigna radiata) from the World Vegetable Gene Bank (Taiwan). BMC Plant Biol..

[45] Ong P.W., Lin Y.P., Chen H.W., Lo C.Y., Burlyaeva M., Noble T., Nair R., Schafleitner R., Vishnyakova M., Bishop-von Wettberg E. (2022). The climatic constrains of the historical global spread of mung bean. bioRxiv.

[46] Christopoulos D.T. (2016). On the Efficient Identification of an Inflection Point. Int. J. Math. Sci. Comput..

[47] Bhardwaj A., Bag S.K. (2019). PLANET-SNP pipeline: PLants based ANnotation and Establishment of True SNP pipeline. Genomics.

[48] Li Q., Yu K. (2008). Improved correction for population stratification in genome-wide association studies by identifying hidden population structures. Genet. Epidemiol..

[49] Kaufman L., Rousseeuw P.J. (2005). Finding Groups in Data: An Introduction to Cluster Analysis.

[50] Zhao Y., Chen F., Zhai R., Lin X., Wang Z., Su L., Christiani D.C. (2012). Correction for population stratification in random forest analysis. Int. J. Epidemiol..

[51] Kursa M.B., Rudnicki W.R. (2010). Feature Selection with the Boruta Package. J. Stat. Softw..

[52] Strumbelj E., Kononenko I. (2010). An Efficient Explanation of Individual Classifications using Game Theory. J. Mach. Learn. Researc.

[53] Molnar C. (2022). Interpretable Machine Learning: A Guide for Making Black Box Models Explainable.

[54] Bayer P.E., Petereit J., Danilevicz M.F., Anderson R., Batley J., Edwards D. (2021). The application of pangenomics and machine learning in genomic selection in plants. Plant Genome.

[55] Stackhouse P.W., Perez R., Sengupta M., Knapp K., Mikovitz J.C., Schlemmer J., Scarino B., Zhang T., Cox S.J. (2016). An Assessment of New Satellite Data Products for the Development of a Long-term Global Solar Resource At 10–100 km. Proceedings of the Solar 2016 Conference.

[56] Dell’Acqua M., Zuccolo A., Tuna M., Gianfranceschi L., Pè M. (2014). Targeting environmental adaptation in the monocot model Brachypodium distachyon: A multi-faceted approach. BMC Genom..

[57] Westengen O.T., Berg P.R., Kent M.P., Brysting A.K. (2012). Spatial Structure and Climatic Adaptation in African Maize Revealed by Surveying SNP Diversity in Relation to Global Breeding and Landrace Panels. PLoS ONE.

[58] Vadez V., Berger J.D., Warkentin T., Asseng S., Ratnakumar P., Rao K.P.C., Gaur P.M., Munier-Jolain N., Larmure A., Voisin A.S. (2012). Adaptation of grain legumes to climate change: A review. Agron. Sustain. Dev..

[59] Van Bel M., Silvestri F., Weitz E.M., Kreft L., Botzki A., Coppens F., Vandepoele K. (2022). PLAZA 5.0: Extending the scope and power of comparative and functional genomics in plants. Nucleic Acids Res..

