# Supplementary material for: Modeling of Flowering Time in Vigna radiata with Artificial Image Objects, Convolutional Neural Network and Random Forest
Source: Plants (Basel). 2022 Dec 1;11(23):3327. doi: 10.3390/plants11233327 (PMC9738219; doi:10.3390/plants11233327)
Supplement: Supplementary file 1 [file plants-11-03327-s001.zip › plants-2037431-supplementary/Figure S7.pdf]

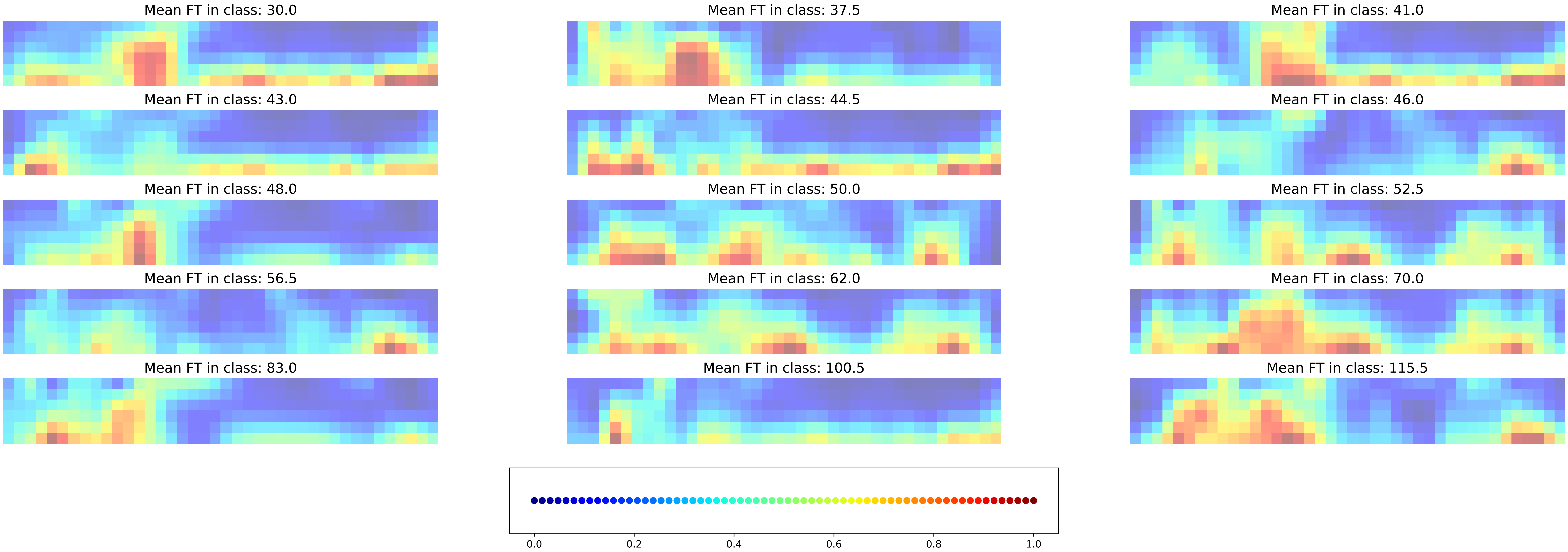
Mean FT in class: 30.0
Mean FT in class: 37.5
Mean FT in class: 41.0
Mean FT in class: 43.0
Mean FT in class: 44.5
Mean FT in class: 46.0
Mean FT in class: 48.0
Mean FT in class: 50.0
Mean FT in class: 52.5
Mean FT in class: 56.5
Mean FT in class: 62.0
Mean FT in class: 70.0
Mean FT in class: 83.0
Mean FT in class: 100.5
Mean FT in class: 115.5
0.0
0.2
0.4
0.6
0.8
1.0
